# Endogenous mouse huntingtin is highly abundant in cranial nerve nuclei, co-aggregates to Abeta plaques and is induced in reactive astrocytes in a transgenic mouse model of Alzheimer’s disease

**DOI:** 10.1186/s40478-019-0726-2

**Published:** 2019-05-20

**Authors:** Maike Hartlage-Rübsamen, Veronika Ratz, Ulrike Zeitschel, Lukas Finzel, Lisa Machner, Janett Köppen, Anja Schulze, Hans-Ulrich Demuth, Stephan von Hörsten, Corinna Höfling, Steffen Roßner

**Affiliations:** 1 Paul Flechsig Institute for Brain Research, University of Leipzig, Leipzig, Germany; 2 Friedrich-Alexander-Universität Erlangen-Nürnberg, Preclinical Experimental Center, Erlangen, Germany; 3 Fraunhofer Institute for Cell Therapy and Immunology, Department of Molecular Drug Design and Target Validation, Halle (Saale), Germany

**Keywords:** Huntingtin, Cranial nerve nuclei, Alzheimer’s disease, Animal model, Amyloid precursor protein, Astrocytes

## Abstract

Pathogenic variants of the huntingtin (HTT) protein and their aggregation have been investigated in great detail in brains of Huntington’s disease patients and HTT-transgenic animals. However, little is known about the physiological brain region- and cell type-specific HTT expression pattern in wild type mice and a potential recruitment of endogenous HTT to other pathogenic protein aggregates such as amyloid plaques in cross seeding events. Employing a monoclonal anti-HTT antibody directed against the HTT mid-region and using brain tissue of three different mouse strains, we detected prominent immunoreactivity in a number of brain areas, particularly in cholinergic cranial nerve nuclei, while ubiquitous neuronal staining appeared faint. The region-specific distribution of endogenous HTT was found to be comparable in wild type rat and hamster brain. In human amyloid precursor protein transgenic Tg2576 mice with amyloid plaque pathology, similar neuronal HTT expression patterns and a distinct association of HTT with Abeta plaques were revealed by immunohistochemical double labelling. Additionally, the localization of HTT in reactive astrocytes was demonstrated for the first time in a transgenic Alzheimer’s disease animal model. Both, plaque association of HTT and occurrence in astrocytes appeared to be age-dependent. Astrocytic HTT gene and protein expression was confirmed in primary cultures by RT-qPCR and by immunocytochemistry. We provide the first detailed analysis of physiological HTT expression in rodent brain and, under pathological conditions, demonstrate HTT aggregation in proximity to Abeta plaques and Abeta-induced astrocytic expression of endogenous HTT in Tg2576 mice.

**Electronic supplementary material:**

The online version of this article (10.1186/s40478-019-0726-2) contains supplementary material, which is available to authorized users.

## Introduction

Huntingtin (HTT) is a 348 kDa protein mainly known for its pathological role in Huntington’s disease (HD). Underlying the autosomal dominantly inherited HD is a pathological increase of CAG repeats in the HTT gene exon 1 that leads to a polyglutamine (polyQ) expansion with *n* > 39 in the corresponding protein [47]. Additionally, the protein contains several cleavage sites for caspases and calpains between amino acids 469 and 586 and fragmentation is suggested to play a significant role in the pathology. As a result, the HTT protein undergoes conformational changes and accumulates in intracellular aggregates [45, 66]. Clinically, the disease is marked by a progressive loss of motoric and cognitive abilities and the selective death of medium spiny neurons in the striatum with a strong correlation between the length of polyQ and the age of disease onset [29, 84, 85].

Since the HTT polyQ expansion is decisive for HD development, most research so far focused on its pathological role and only little is known about the physiological function of HTT [9, 50]. The protein is ubiquitously expressed in both rodents and humans with the highest level in the brain [88]. The mouse Htt gene is located on chromosome 5 and shows 86% homology to the human gene and 91% homology to the protein sequence. The main difference is reflected by the physiological presence of only six CAG repeats in mice in comparison to the mean length of 20–22 CAGs in unaffected human individuals [2]. Systemically, physiological HTT appears to play a significant role in brain development during embryogenesis, as knock-out mice die at an early embryonic stage [19]. Subcellularly, the physiological form of HTT is mainly localized to the cytoplasm, but some studies report presence of HTT or HTT fragments in the nucleoplasm as well [4, 17, 37, 64, 70]. However, so far detailed information is neither available on the physiological region-specific expression pattern of endogenous HTT in mouse brain nor on potential interactions of HTT with other pathogenic and aggregation-prone proteins and was therefore investigated in the present study.

Neurodegenerative diseases like HD [45, 66], but also Alzheimer’s disease (AD) [26] and Parkinson’s disease (PD) [61] share the characteristics of protein misfolding followed by aggregation. Traditionally, basic and clinical research focused on the disease-defining proteins HTT in HD, Abeta and Tau in AD and α-synuclein in PD, respectively, to reveal underlying mechanism of pathogenic protein aggregation and its impact on initiation and progression of disease.

However, post mortem examinations of patients revealed co-existence of different misfolded proteins in brain tissue, raising the question whether there might be overlaps and analogies between the specific pathologies [16, 21, 31, 33]. Given the fact that proteins can act as homologous seeds [11, 41, 51], the question arose whether seeding could also take place in a heterologous manner. For instance, in vitro and in vivo evidence suggests cross talk between the Abeta peptide and prion protein as well as tau protein [28, 59]. This scenario, however, is not limited to the co-aggregation of different peptides affected in a particular disease, but also applies to co-aggregation of proteins characteristic for different clinical entities suggesting cross-disease mechanisms of pathogenic protein co-aggregation events.

In particular, the relationship between aggregating peptides typical for AD and PD has been studied before. For example, it was demonstrated that α-synuclein can initiate tau aggregation [25]. Additionally, the molecular interrelation of α-synuclein with Abeta was investigated, demonstrating specific sites of interaction between membrane-bound α-synuclein with Abeta and vice versa [52]. In mutant α-synuclein transgenic mice, α-synuclein overexpression-induced neurodegeneration led to accumulation of insoluble mouse Abeta [23]. Also, in human PD brain cortical α-synuclein load is associated with Abeta plaque burden [45]. In a double transgenic mouse model Abeta was shown to promote α-synuclein aggregation and toxic conversion in vivo, suggesting that abnormal interactions between misfolded proteins might contribute to disease pathogenesis [55]. In the brains of patients with AD/PD and in transgenic mice, Abeta and α-synuclein were shown to co-immunoprecipitate and to form complexes [80]. Additionally, when aspects of AD and PD pathologies are combined in transgenic mice, Abeta, tau and α-synuclein pathologies are enhanced and cognitive decline is accelerated [12].

In contrast, no information is currently available about the potential cross seeding of Abeta and HTT in AD or HD-associated conditions. Therefore, in order to study a potential influence of Abeta pathology on HTT expression and accumulation we used the transgenic mouse model Tg2576. These mice overexpress human APP695 with the Swedish double mutation KM670/671NL and thereby reproduce the Abeta pathology in an age-dependent manner starting around 11 to 13 months of age [34, 39, 42].

## Materials and methods

### Animals

The expression of endogenous HTT in brains of 3-month-old wild type animals was analyzed in the inbred mouse lines C57BL/6 N (*N* = 3) and BALB/c (*N* = 3) and in the outbred line CD1 (*N* = 3) as well as in Wistar rats (*N* = 3) and hamster (*N* = 1). In order to reveal a potential influence of amyloid pathology on endogenous HTT expression in brain, human APP-transgenic Tg2576 mice and wild type littermates at postnatal ages of 15, 18, 24, 28 and 32 months were analyzed. Tg2576 mice are on C57BL/6xSJL background and express human APP695 carrying the Swedish mutation KM670/671NL driven by the hamster prion protein promoter [39]. Founder mice were provided by K. Hsiao-Ashe. Animals were housed at 12 h day/12 h night cycles with food and water ad libitum in cages that contained red plastic houses (Tecniplast, Hohenpeißenberg, Germany) and shredded paper flakes to allow nest building. All experimental protocols were approved by Landesdirektion Sachsen, license numbers T11/16 (rat), T14/16 (hamster) and T28/16 (mouse) and all methods were carried out in accordance with the relevant guidelines and regulations.

### Immunohistochemistry

#### Tissue preparation

Mice, rats and hamster were sacrificed by CO_2_ inhalation and perfused transcardially with 0.9% saline followed by perfusion with 4% paraformaldehyde in phosphate buffer (0.1 M, pH 7.4). The brains were removed from the skull and post-fixed by immersion in the same fixative overnight at 4 °C. After cryoprotection in 30% sucrose in 0.1 M phosphate buffer for three days, horizontal and coronal sections, respectively, of 30 μm were cut on a sliding microtome and collected in phosphate buffer with sodium azide.

#### Single labelling of HTT

In order to detect HTT, single labelling immunohistochemistry was performed on free floating brain sections of C57BL/6 N, CD1 and BALB/c mice, Wistar rat and hamster. To allow for a complete mapping of endogenous HTT expression, series of horizontal sections (one in eight) and of coronal sections (one in six) were used for staining. Brain sections were washed in phosphate buffer for 5 min and endogenous peroxidases were inactivated by treating brain slices with 60% methanol containing 1% H_2_O_2_ for 30 min followed by three washing steps with Tris buffered saline (TBS, 0.1 M, pH 7.4) for 5 min each. After masking unspecific binding sites with blocking solution (5% normal donkey serum in TBS containing 0.3% Triton X-100) for 30 min sections were incubated with the primary rabbit monoclonal anti-HTT antibody (EP867Y, raised against HTT amino acids 550–650; Abcam). In initial experiments brain sections were incubated with the HTT antibody at dilutions of 1:500, 1:1000, 1:2000 and 1:4000 overnight at 4 °C. Brain sections were washed three times in TBS for 5 min each before being incubated with biotinylated, secondary donkey anti-rabbit antibodies (Dianova; 1:1000) in TBS containing 2% bovine serum albumin (BSA) for 60 min. After three washing steps in TBS for 5 min each, slices were incubated with ExtrAvidin peroxidase (Sigma; 1:2000) in TBS/2% BSA. After washing steps, slices were pre-incubated in Tris buffer (0.05 M, pH 7.6) for 5 min before visualization of peroxidase binding was performed by incubation with 4 mg 3,3′-diaminobenzidine (DAB) and 2.5 μl H_2_O_2_ per 5 ml Tris buffer. After washing, sections were mounted onto glass slides and coverslipped. Based on the results of the different primary antibody dilutions (Additional file 1: Figure S1a) the respective serial stainings were done with a 1:4000 dilution of the primary HTT antibody. The specificity of the EP867Y was validated by the detection of much stronger HTT immunoreactivity in brains of HTT transgenic BACHD mice [30] (Additional file 1: Figure S1b).

#### Double and triple immunofluorescent labellings

In order to reveal the brain region- and cell type-specific HTT labelling, the rabbit anti-HTT monoclonal antibody was applied in cocktails with primary antibodies against choline acetyltransferase (ChAT), dopamine and cAMP regulated phosphoprotein 32 kDa (DARPP-32), urocortin-1 (Ucn-1), tyrosine hydroxylase (TH), the astrocyte marker glial fibrillary acidic protein (GFAP) and the microglial marker ionized calcium-binding adapter molecule 1 (Iba-1) as specified in Table 1. In addition, a possible association of HTT with amyloid deposits in APP-transgenic Tg2576 mice was investigated by double labelling of HTT with an antibody directed against Abeta (4G8) and combined with a fluorescent histological stain for fibrillary amyloid (Thioflavin S, ThS), respectively (Table 1). Brain sections were incubated with cocktails of primary antibodies overnight at 4 °C. On the next day, sections were washed three times with TBS and were then incubated with cocktails of Cy2-, Cy3- or Cy5-conjugated donkey anti-mouse, -rabbit or -goat antisera (1:400 each, Dianova) for 60 min at room temperature. After washing, sections were mounted onto glass slides and coverslipped. ThS staining was performed on mounted sections, by briefly hydrating sections in *aqua dest*., incubation in ThS (1%) for 20 min and differentiation in 80% ethanol for 20 to 40 min, followed by two rinses in *aqua dest*., air drying and coverslipping.

**Table 1:** Cocktails of antibodies used for double and triple labeling immunohistochemistry

Primary antibody/stain	Dilution	Host	Company	Secondary antibody
HTT (EP867Y)	1:1000	rabbit	Abcam	donkey anti-rabbit Cy2
ChAT	1:100	goat	Millipore	donkey anti-goat Cy3
HTT (EP867Y)	1:1000	rabbit	Abcam	donkey anti-rabbit Cy2
DARPP-32	1:200	guinea pig	Syn. Systems	donkey anti-guinea pig Cy3
HTT (EP867Y)	1:1000	rabbit	Abcam	donkey anti-rabbit Cy2
Ucn-1	1:100	goat	Santa Cruz	donkey anti-goat Cy3
HTT (EP867Y)	1:1000	rabbit	Abcam	donkey anti-rabbit Cy2
TH	1:50	goat	Santa Cruz	donkey anti-goat Cy3
HTT (EP867Y)	1:1000	rabbit	Abcam	biotin-donkey anti-rabbit + SA-HRP
ThS	1%	-	Sigma	-
HTT (EP867Y)	1:1000	rabbit	Abcam	donkey anti-rabbit Cy3
Abeta (4G8)	1:2000	mouse	Biolegend	donkey anti-mouse Cy2
HTT (EP867Y)	1:1000	rabbit	Abcam	donkey anti-rabbit Cy3
Iba1	1:200	guinea pig	Syn. Systems	donkey anti-mouse Cy5
HTT (EP867Y)	1:1000	rabbit	Abcam	donkey anti-rabbit Cy3
GFAP (G-A-5)	1:1000	mouse	Sigma	donkey anti-mouse Cy5
HTT (EP867Y)	1:1000	rabbit	Abcam	donkey anti-rabbit Cy3
ThS	1%	-	Sigma	-
GFAP (G-A-5)	1:1000	mouse	Sigma	donkey anti-mouse Cy5

Secondary antibodies were all from Dianova and used at a dilution of 1:400

*HTT* huntingtin, *ChAT* choline acetyltransferase, *DARPP-32* dopamine and cAMP regulated phosphoprotein 32 kDa, *Ucn-1* Urocortin-1, *TH* tyrosine hydroxylase, *SA-HRP* horseradish peroxidase-conjugated streptavidin, *Iba1* ionized calcium-binding adapter molecule 1, *ThS* ThioflavinS, *GFAP* glial fibrillary acidic protein

For all single and double immunohistochemical labellings in brain sections described above and for immunocytochemistry of primary neuronal and glial cell cultures described below, control experiments in the absence of primary antibodies were carried out. In each case, this resulted in unstained brain sections or primary cells, respectively (not shown). In addition, switching the fluorescent labels of the secondary antibodies (i.e. detection of HTT by secondary donkey anti-rabbit-Cy3 and visualization of ChAT by donkey anti-goat-Cy2) generated similar results to the procedure outlined above (not shown).

### Microscopy

#### Light microscopy

Brain tissue sections of wild type and APP-transgenic Tg2576 mice immunochemically stained with DAB for HTT expression were examined with an Axio-Scan.Z1 microscope connected with a Colibri.7 light source and an Axiocam 506 camera (Carl Zeiss, Göttingen, Germany). Images of complete brain sections were taken using a 10x objective lens with 0.45 numerical aperture (Zeiss). The ThS counterstaining was revealed in the 488 nm fluorescence channel of the microscope. Images were digitized by means of ZEN 2.3 software and exported with the NetScope program (Net-Base Software GmbH, Freiburg, Germany) where regions of interest were excised.

#### Confocal laser scanning microscopy

Laser scanning microscopy (LSM 510, Zeiss, Oberkochen, Germany) using an Axioplan2 microscope was performed to reveal co-localization of HTT with the neuronal markers ChAT, DARPP-32, TH, Ucn-1, with glial markers GFAP and Iba-1 and with Abeta deposits. For Cy2-labelled antigens (green fluorescence), an argon laser with 488 nm excitation was used and emission from Cy2 was recorded at 510 nm applying a low-range band pass (505–550 nm). For Cy3-labelled antigens (red fluorescence), a helium-neon-laser with 543 nm excitation was applied and emission from Cy3 at 570 nm was detected applying high-range band pass (560–615 nm) and Cy5-labelled antigens (blue fluorescence) were detected using excitation at 650 nm and emission at 670 nm. Images of areas of interest were taken using a 20x objective lens with 0.75 numerical aperture (Zeiss). Photoshop CS2 (Adobe Systems, CA) was used to process the images obtained by light and confocal laser scanning microscopy. Care was taken to apply the same brightness, sharpness, color saturation and contrast adjustments in the various pictures.

### Huntingtin aggregation assays

#### Expression and purification of huntingtin

The expression vector pGEX-6P-1 containing exon-1 of HTT and encoding 52 glutamine residues (HTTQ52) was kindly provided by Zoya Ignatova, University of Hamburg, and used to express recombinant HTT for aggregation assays. HTTQ52 with N-terminal GST-tag followed by a polyhistidine tag was expressed in *E. coli* strain BL21 after induction with 400 μM IPTG at 24 °C for 4 h. After harvesting by centrifugation, cells were disrupted by enzymatic lysis and sonification. The first purification step was carried out through Ni^2+^-chelating chromatography on a Streamline Chelating resin (Streamline Chelating, GE Healthcare Life Sciences). Fractions containing the expression construct were further purified in a second step via a glutathione sepharose resin (Glutathione Sepharose 4FF, GE Healthcare Life Sciences). The removal of glutathione was achieved by dialysis overnight against buffer containing 50 mM Tris/HCl, 150 mM KCl, pH 8.5. Fractions of interest were lyophilized and stored at − 20 °C until usage. The purity of the samples was assessed by SDS-PAGE and mass spectrometry. Protein concentrations were determined using UV absorption at 280 nm.

#### Synthesis of amyloid peptides

Synthesis and purification of Abeta (1–42) was performed by solid-phase syntheses as described previously [68]. Structures and purities of the Abeta peptide were confirmed by mass spectrometry. The lyophilized peptide was dissolved in HFIP (Sigma-Aldrich, St. Louis, USA) overnight. The solvents were evaporated under a stream of nitrogen and Abeta peptides were dissolved in 0.2 M NaOH, followed by buffer and finally titrated with 0.2 M HCl.

#### ThT fluorescence kinetics assay

The thioflavin T (ThT) assay was carried out as described previously [67]. The fibrillation process was induced by cleavage of the GST and polyhistidine tag by PreScission protease (GE Healthcare Life Sciences) and monitored by ThT fluorescence (20 μM, Sigma-Aldrich) at λex = 440 nm and λem = 490 nm in aggregation buffer (50 mM Tris/HCl, 50 mM KCl, 4 mM MgCl_2_, pH 8.0) on a FluoStar Optima plate reader (BMG Labtech, Ortenberg, Germany). Signals were recorded at 30 °C under continuous shaking (300 rpm). Data analysis was done according to [38]. In brief, lag times (t_lag_) of the aggregation were determined by fitting the straight lines *a* to the baseline of the lag phase and *b* as a tangent to the steepest region of the growth phase curve. T_lag_ is defined as the time point where the two lines *a* and *b* intersect. To obtain the aggregation rate k, the growth phase was fitted to the function: y = A + B*exp(−*kx*). Values were obtained from 3 independent determinations and displayed as mean ± S.D.

#### Transmission electron microscopy and immunogold labelling

For immunogold staining the fibril samples (5 μl) were fixed on formvar carbon-coated copper grids (Plano, Wetzlar, Germany) with 2% (w/v) paraformaldehyde (Merck, Darmstadt, Germany) in 0.1 M HEPES buffer (Thermo Fisher, Waltham, USA) for 20 min followed by washing with distilled water. The sections were next incubated for 30 min at RT with blocking solution (1% (w/v) Bovine Serum Album (BSA, Sigma-Aldrich) with 0.1% Tween-20 (Carl Roth, Karlsruhe, Germany) in 0.1 M HEPES buffer) to prevent unspecific binding. After blocking, the grids were incubated overnight with the primary antibodies directed against HTT (sheep polyclonal, S830, kindly provided by Gillian P. Bates, London, UK; 1:500) and 6E10 (mouse monoclonal, recognizes Abeta (1–42), Merck; 1:250) diluted in blocking solution. After 4 washes of 5 min with blocking solution the grids were incubated for 90 min with secondary antibodies coupled to colloidal gold: anti-sheep IgG 4 nm gold (donkey polyclonal, Jackson ImmunoResearch, Cambridgeshire, UK) and anti-mouse IgG 20 nm gold (goat polyclonal, Abcam), 1:20 diluted in blocking solution. After three washing steps with distilled water the grids were negative stained for 5 min with 2% (v/v) uranyl acetate (SERVA Electrophoresis GmbH, Heidelberg, Germany). Transmission electron microscopic images were recorded using a Zeiss EM 912 Omega electron microscope operating at 80 kV.

### Primary neuronal and astrocytic cultures

Primary neuronal and astroglial cultures were established from APP-transgenic Tg2576 mice and wild type littermates and grown under standard conditions [35]. Briefly, primary neuronal cell cultures were derived from fetal mouse brain at gestation day 16. Astrocyte-rich primary cell cultures were derived from brains of newborn mice. Cells were grown in 24 well plates on poly-L-lysine-coated coverslips and maintained in DMEM-based medium at 37 °C in a humidified atmosphere with 95% air/5% CO_2_. The purity of primary cultures was verified by RT-PCR and by immunocytochemistry against neuronal and astrocytic markers (Additional file 1: Figure S2).

### RNA isolation and real time quantitative PCR

A real time quantitative polymerase chain reaction (RT-qPCR) with RNA from primary neurons and astrocytes derived from wild type and Tg2576 mouse brains, respectively, was performed to analyze cell type-specific HTT expression. RNA of cultivated wild type and Tg2576 neurons and astrocytes was isolated using the Trizol RNA isolation protocol [10]. Quality and concentration of RNA was analyzed with the photometer NanoDrop 2000 at wavelengths 260 nm and 280 nm. Based on the results obtained from primer testing (Additional file 1: Figure S3a), two primer pairs (#1 and #4) were chosen to analyze the HTT expression in cultivated primary astrocytes and neurons. Primer pair #1 is directed against a region spanning HTT exon 1 and 2, whereas primer pair #4 recognizes a region located more carboxy-terminally corresponding to the epitope of HTT antibody used in this study. A one-step RT-qPCR was performed to analyze expression of HTT. All working steps were performed on ice and Qiagen RT-PCR Kit was used. RNA of three wild type and three Tg2576 cell preparations were diluted in RNase free water to obtain a 20 ng/μl solution. All diluted RNA samples were denatured in the Rotor GeneTM 6000 Cycler at 70 °C for 5 min to dissolve possible secondary structures and were stored on ice until further usage. Cyclophilin A (CycA) was used as reference gene. Primers for CycA [81] and HTT (50 μM each) were diluted in *aqua dest*. to obtain a concentration of 10 μM. Of all samples 0.5 μl RNA were added to 9.5 μl of master mix and the RT-qPCR was performed with a Rotor GeneTM 6000 Cycler according to Additional file 1: Table S1. Specificity of PCR products was verified by electrophoresis and by analysis of the obtained C_T_ values. A detailed analysis of threshold cycle (CT) results obtained with RT-qPCR was performed with the ΔΔCT method for relative quantification. The linearity of results obtained was validated in a standard curve (Additional file 1: Figure S3b) and PCR control experiments without RT and after DNAse treatment are shown in Additional file 1: Figure S3c.

### Immunocytochemistry

To determine the cell type-specific expression of endogenous HTT in primary neurons and astrocytes double immunofluorescent labellings of HTT and cell type-specific markers were performed as described for immunohistochemistry on brain sections. Finally, coverslips were air-dried, embedded in entellan/toluene on microscopic slides and stored at 4 °C in the dark. Additionally, double labelling with neuron- and astrocyte-specific antibodies was performed on neuronal and astroglial primary cultures, to examine the identity and purity of the respective cell cultures.

## Results

### Expression of endogenous HTT in wild type mouse brain

First, the expression of endogenous HTT in C57BL/6 N mouse brain was analyzed by immunohistochemistry. HTT immunoreactivity was present at low levels throughout the brain but was highly enriched in a number of morphologically clearly defined neuronal nuclei. The identities of these nuclei brought out by HTT labelling were determined according to their morphology as well as to their rostro-caudal and dorso-ventral position using a mouse brain atlas [22].

**Fig. 1:**
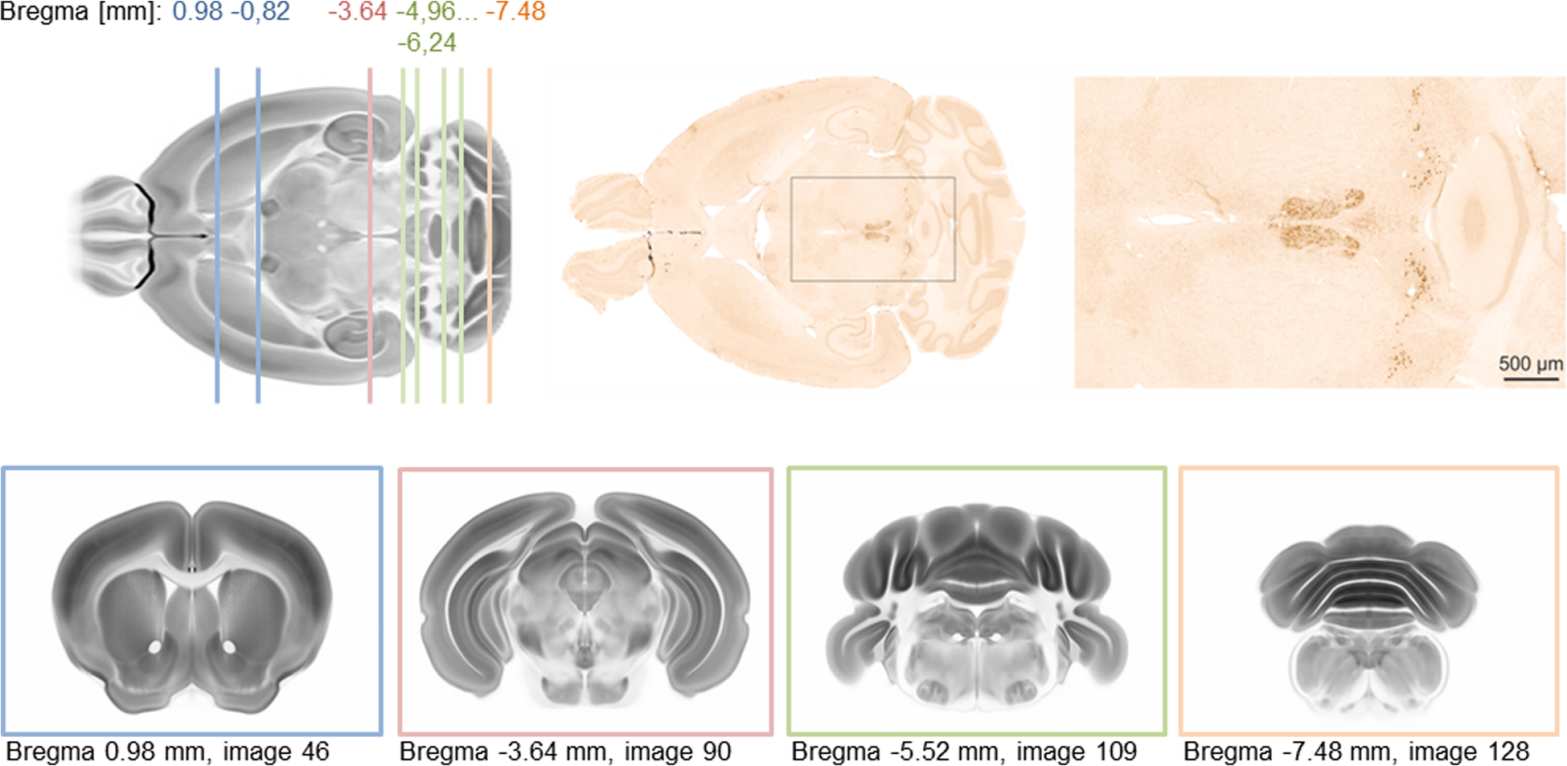
Immunohistochemistry for huntingtin (HTT) on mouse horizontal brain sections (top, middle and right) and schematic presentation of coronal cutting levels with high abundance of strongly HTT immunoreactive neurons. Note the enrichment of HTT immunoreactivity in caudal brain structures. The anatomical templates used to illustrate cutting levels are shape and signal intensity averages from the Allen Mouse Brain Connectivity Atlas [60]

At a high concentration of the primary antibody, a wide-spread HTT immunoreactivity was detected throughout the mouse brain. When diluting the HTT primary antibody from 1:500 to 1:4000, only neuronal populations with highly abundant HTT expression were detected (Additional file 1: Figure S1a). Applying a high HTT antibody dilution to horizontal mouse brain sections, a significant number of neuronal populations in caudal brain regions were still strongly HTT immunoreactive while ubiquitous neuronal staining was abolished (Fig. 1).

**Fig. 2:**
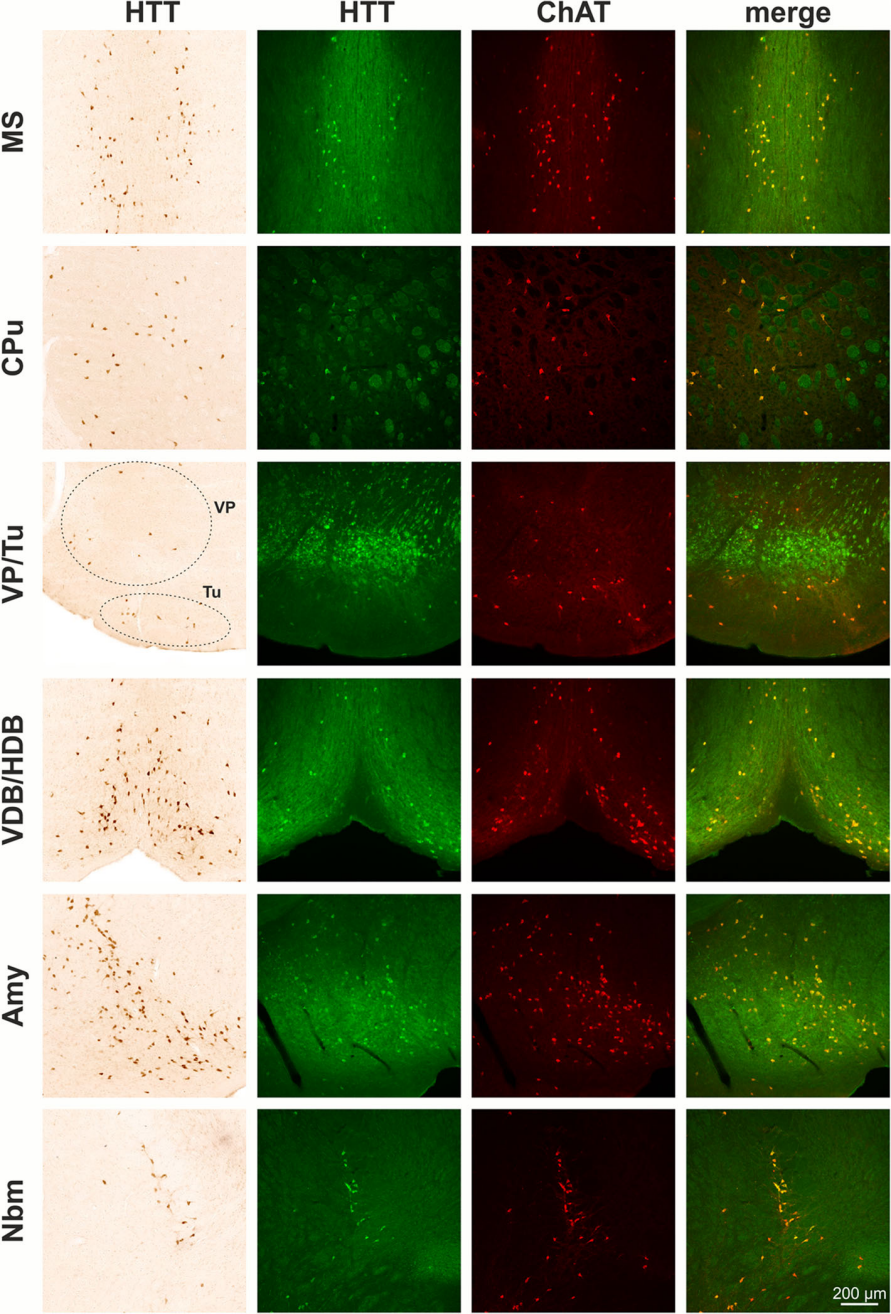

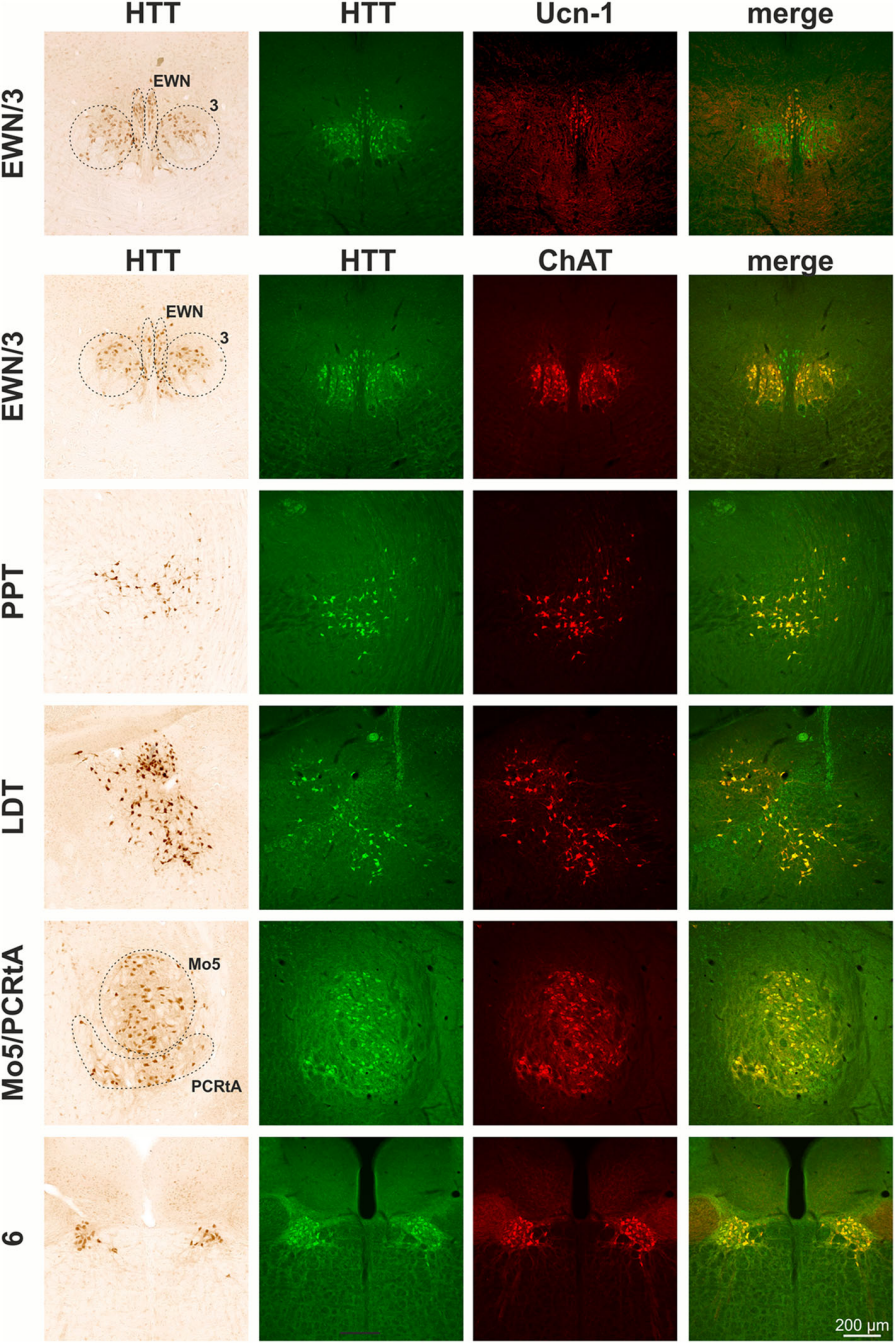

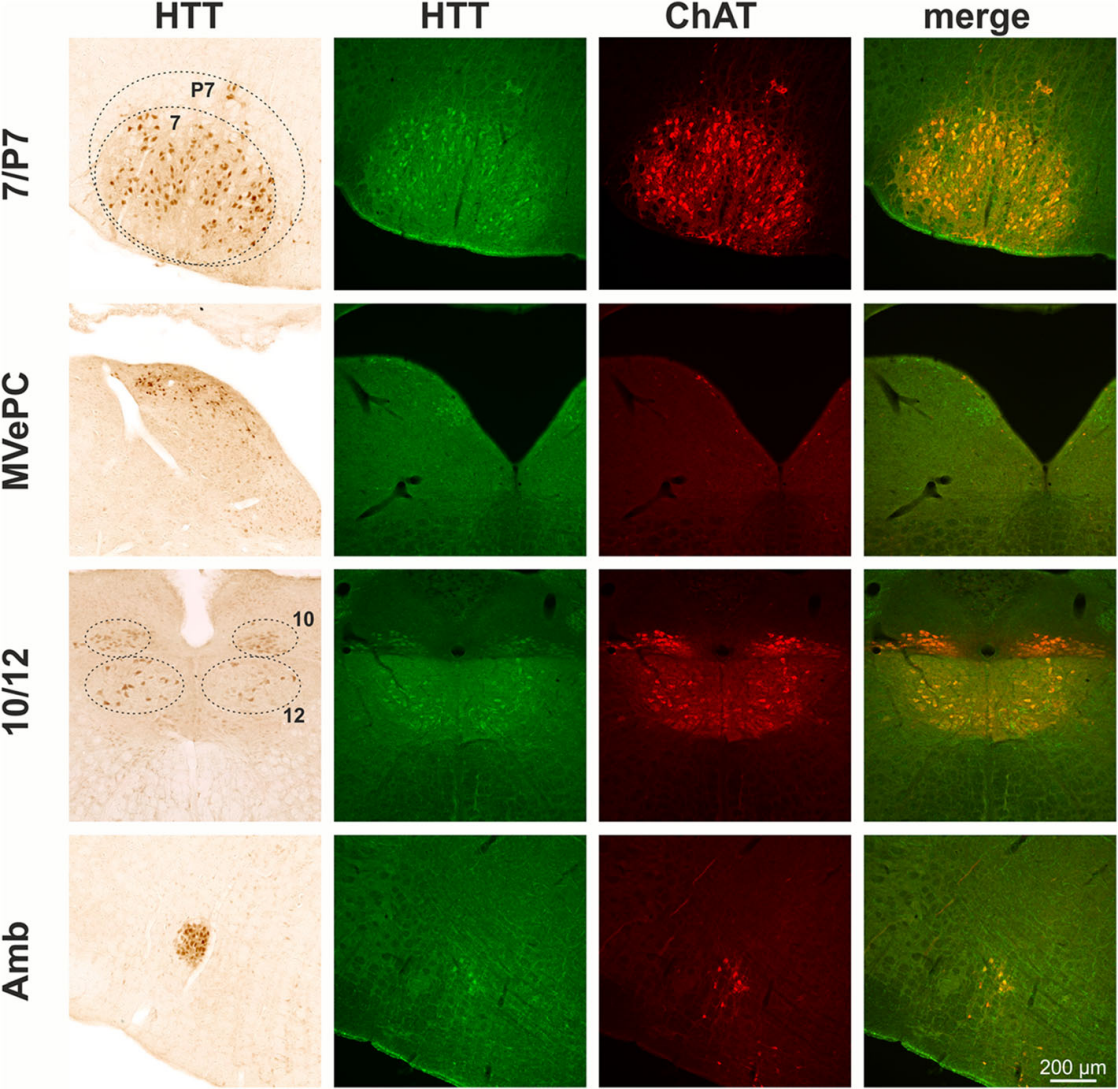
Immunohistochemical detection of huntingtin (HTT) in mouse brain. HTT is highly enriched in cholinergic striatal neurons (CPu), cholinergic neurons of the basal forebrain including medial septum (MS), vertical and horizontal diagonal band (VDB/HDB) and nucleus basalis magnocellularis (Nbm) as well as in cholinergic neurons of the olfactory tubercle (Tu), ventral pallidum (VP), and amygdala (Amy). At more caudal coronal brain levels, distinct HTT immunoreactivity present in cholinergic (oculomotor nerve nucleus; 3) and in urocortin-1 positive neurons of the Edinger-Westphal nucleus (EWN), pedunculopontine tegmental nucleus (PPT), laterodorsal tegmental nucleus (LDT), motor trigeminal nucleus (Mo5), nucleus abducens (6), nucleus facialis (7) and perifacial zone (P7), the parvicellular medial vestibular nucleus (MVePC), nucleus vagus (10), the hypoglossal nucleus (12) and nucleus ambiguus (Amb)

The screening of every 6th coronal section identified HTT immunoreactive neurons at different coronal levels. These populations include a distinct subpopulation of striatal neurons, neurons in the basal forebrain including medial septum (MS), vertical and horizontal diagonal band (VDB/HDB), ventral pallidum (VP), olfactory tubercle (Tu), nucleus basalis magnocellularis (Nbm) as well as amygdala (Amy) (Fig. 2). At more caudal coronal brain levels, significant HTT immunoreactivity was detected in Edinger-Westphal nucleus (EWN), pedunculopontine tegmental nucleus (PPT), laterodorsal tegmental nucleus (LDT), motor trigeminal nucleus (Mo5), nucleus abducens (6), nucleus facialis (7) and the perifacial zone (P7), the parvicellular medial vestibular nucleus (MVePC), nucleus ambiguus (Amb), nucleus vagus (10) and the hypoglossal nucleus (12) (Fig. 2).

Interestingly, EP867Y detected HTT immunoreactivity is strongly enriched in cranial nerve nuclei and also significantly present in basal forebrain and in a subset of striatal neurons. In order to validate the identity of the HTT-positive neuronal nuclei and subpopulations, respectively, double labellings with ChAT, Ucn-1, DARPP-32, a marker for medium spiny neurons, and TH were performed. As a result, almost all heavily stained HTT-positive nuclei were found to be cholinergic which was evident by ChAT co-expression. This demonstrates HTT expression by neurons of the respective cranial nerve nuclei and by cholinergic neurons of the basal forebrain (MS, VDB/HDB and Nbm) as well as cholinergic interneurons of the striatum (Fig. 2). In contrast, HTT immunoreactivity was absent from DARPP-32-positive striatal medium spiny neurons and from TH-positive locus coeruleus neurons neighboring the LDT (not shown). The only non-cholinergic neuronal population showing high HTT immunoreactivity in mouse brain was the Ucn-1-positive neuronal subregion of EWN (Fig. 2). For a general overview, the HTT immunoreactive neuronal populations along the rostro-caudal extension of the brain and their attribution to cranial nerve nuclei are summarized in Table 2.

**Table 2:** Neuronal populations with highly abundant HTT expression

Bregma [mm]	structure name		Association with cranial nerve nuclei	
+ 0.98	MS	medial septum nucleus		
	CPu	caudate putamen		
	VP/Tu	ventral pallidum olfactory tubercle	I	Nervus olfactorius
	VDB/HDB	nucleus ventral limb diagonal band		
−0.82	Amy	amygdaloid area		
	Nbm	Nucleus basalis magnocellularis		
−3.64	EWN	Edinger Westphal nucleus		
	3	oculomotor nerve nucleus	III	Nervus oculomotorius
−4.96	PPT	pedunculopontine tegmental nucleus		
	LDT	laterodorsal tegmental nucleus		
−5.34	Mo5/PCRtA	motor trigeminal nucleus parvicellular reticular nucleus		
−5.80	6	abducens nucleus	VI	Nervus abducens
	7/P7	facial nucleus/perifacial zone	VII	Nervus facialis
−6.24	MVePC	medial vestibular nucleus parvocellular	VIII	Nervus vestibulocochlearis
−7.48	10	dorsal motor nucleus vagus	X	Nervus vagus
	12	hypoglossal nucleus	XII	Nervus hypoglossus
	Amb	ambiguus nucleus		

**Fig. 3:**
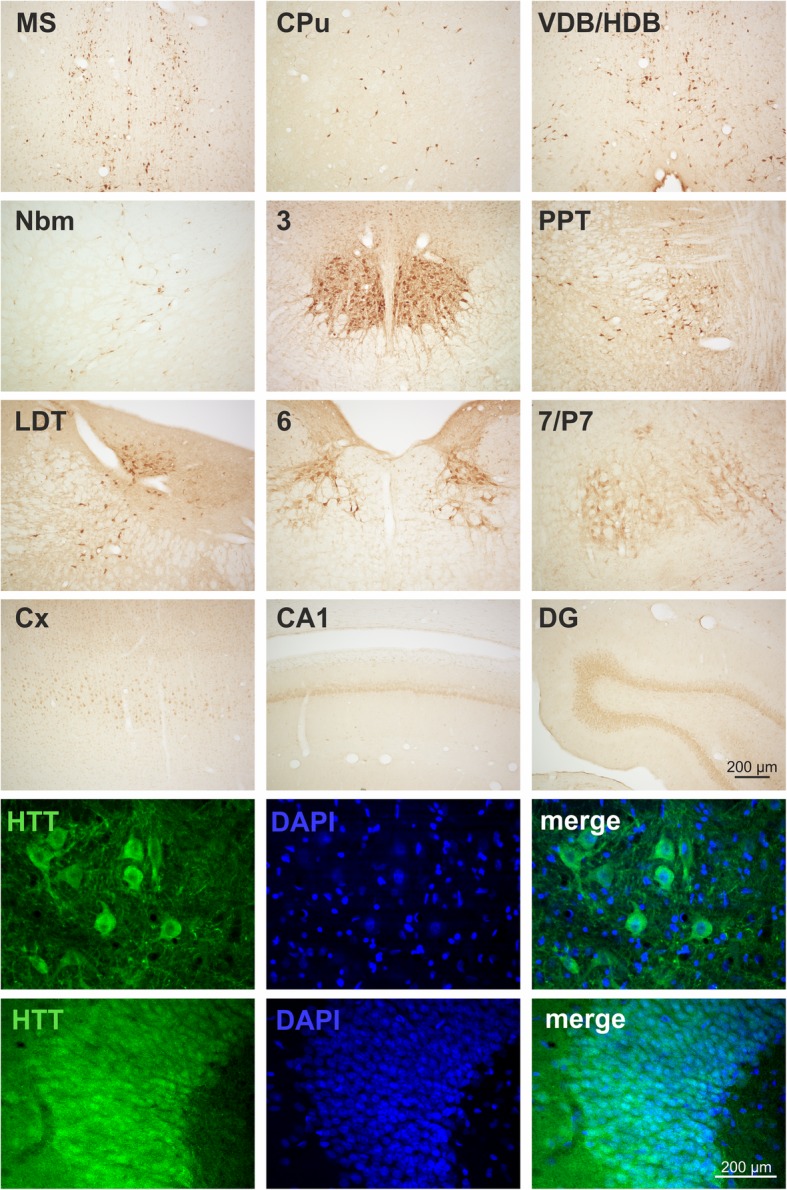
Immunohistochemical HTT labelling in coronal brain sections of Wistar rat. Note the high abundance of HTT immunoreactivity in medial septum (MS), caudate putamen (CPu), vertical and horizontal diagonal band (VDB/HDB), nucleus basalis magnocellularis (Nbm), oculomotor nerve nucleus (3), pedunculopontine tegmental nucleus (PPT), laterodorsal tegmental nucleus (LDT), nucleus abducens (6), nucleus facialis (7) and perifacial zone (P7). In addition and in contrast to mouse brain, significant HTT immunoreactivity was detected in cortical pyramidal neurons (Cx), and in cornu ammonis 1 (CA1) pyramidal and dentate gyrus (DG) granule neurons of the hippocampus

Since this is the first detailed analysis of endogenous HTT expression in mouse brain, we considered it important to validate our observations made in the C57BL/6 N strain in brains of two other mouse lines (CD1 and BALB/c). In the brains of these mouse lines very similar HTT expression patterns were observed (not shown). Also in Wistar rat (Fig. 3) and in hamster (Additional file 1: Figure S4) as different rodent species there was an enrichment of HTT immunoreactivity in cranial nerve nuclei and in the cholinergic basal forebrain and caudate putamen structures. A distinct difference to the HTT immunoreactivity in mouse brain, however, was the labelling of neocortical neurons, in particular in layer III pyramidal neurons, CA1 pyramidal neurons and dentate gyrus granule neurons (Fig. 3; Additional file 1: Figure S4), which was not depicted in mouse brain using the same HTT antibody dilution. However, the cortical and hippocampal neurons immunoreactive for HTT in rat brain did not display a distinct cytoplasmic labelling typical for basal forebrain and cranial nerve nuclei, but rather a nuclear HTT localization. The presence of HTT in neuronal nuclei is exemplarily shown for dentate gyrus granule cells (Fig. 3). However, most neuronal populations in basal forebrain and in cranial nerve nuclei displayed cytoplasmic/neuritic labelling (Fig. 3).

### HTT expression and aggregation to Abeta plaques in APP-transgenic Tg2576 mice

At sites of endogenous HTT expression, the formation of cytotoxic HTT aggregates is likely when HTT variants with expanded CAG repeats are expressed. However, in different clinical conditions associated with pathogenic protein aggregation in brain, the formation of protein co-aggregates generated in cross-seeding events is reported.

**Fig. 4:**
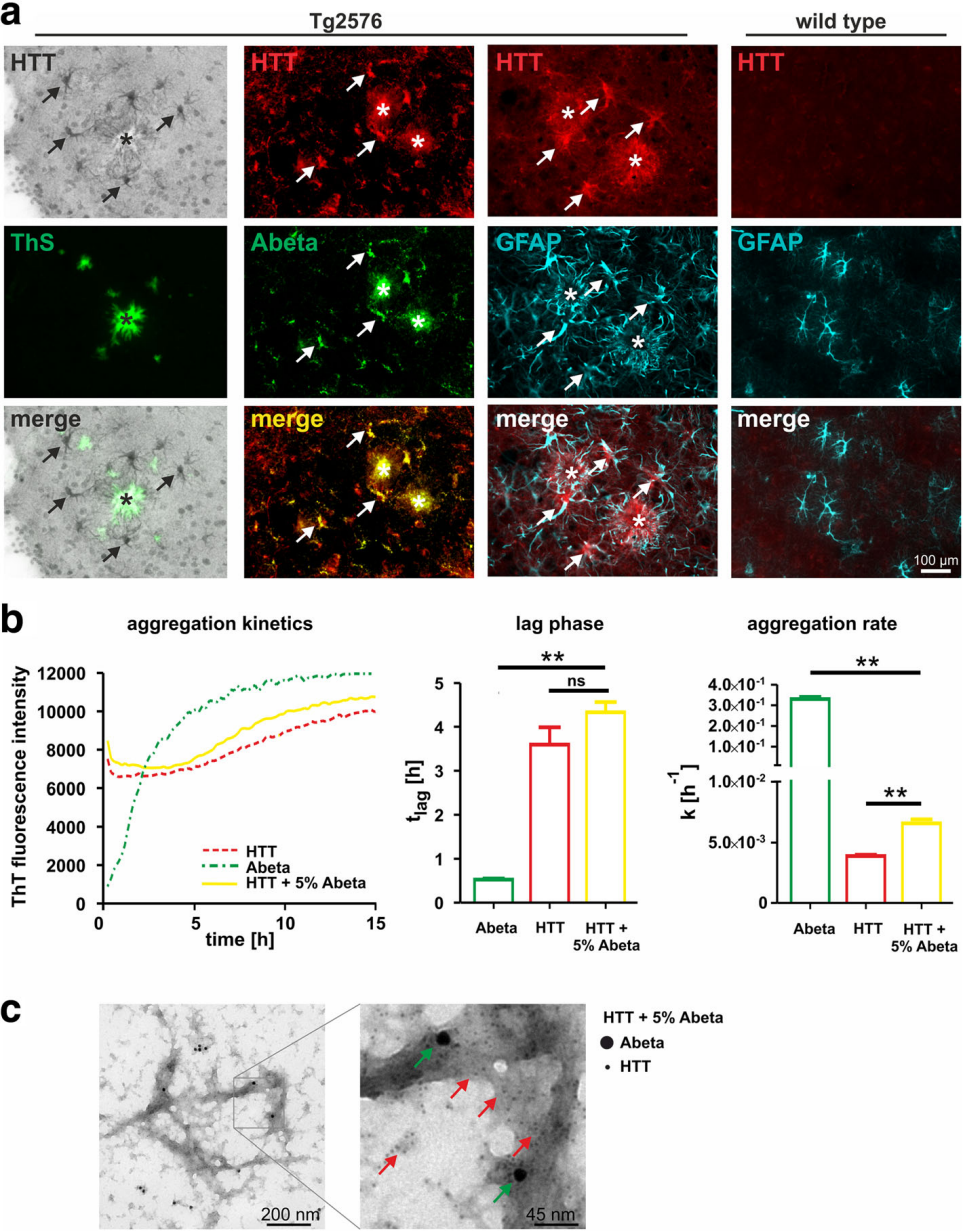
**a** Association of HTT with Abeta plaques and presence in reactive astrocytes in entorhinal cortex of an 18-month-old Tg2576 mouse. HTT immunoreactivity was found to be present in Abeta plaque-associated glia-like structures (Tg2576 left row, arrows) and aggregate-like structures in the periphery of ThS-positive Abeta plaques (Tg2576 left row, asterisk). This correlation can also be shown using the pan-Abeta-specific antibody 4G8 (Tg2576 middle row). Double fluorescence labelling of HTT and GFAP demonstrates the presence of HTT in reactive astrocytes (arrows) surrounding Abeta plaques (Tg2576 right row, asterisk). No glial HTT immunoreactivity or aggregate-like structures were detected in age-matched wild type mouse entorhinal cortex (wild type row). **b** Aggregation of 22.5 μM HTT (red dotted line) and its stimulation by addition of 1 μM Abeta (1–42) (yellow solid line) monitored by ThT fluorescence. Note the typical aggregation curve for 1 μM Abeta (1–42) aggregation alone (green broken line). Statistical analysis revealed an accelerated HTT aggregation rate (right diagram) without effects on the lag phase (middle diagram) after addition of small amounts of Abeta (1–42). **c** Electron microscopic demonstration of the co-occurrence of HTT (4 nm gold particles) and Abeta (1–42) (20 nm gold particles) after immunogold labelling of fibrils generated from 22.5 μM HTT in the presence of 1 μM Abeta (1–42)

In order to investigate potential protein cross seeding of endogenous mouse HTT by Abeta aggregates, we analyzed the HTT immunoreactivity in brains of 18-month-old APP-transgenic Tg2576 mice with amyloid pathology. Basically, the general HTT staining pattern in Tg2576 mouse brain reflected the labelling detected in wild type mice (not shown). However, neocortical and hippocampal HTT immunoreactivity in aged Tg2576 mice appeared to be additionally associated with amyloid plaque pathology. Therefore, a series of double labellings of HTT with (i) ThS, (ii) the Abeta antibody 4G8 and (iii) the astrocyte marker GFAP were performed and analyzed by confocal laser scanning microscopy (Fig. 4a). These double immunofluorescent stainings demonstrated a punctate HTT immunoreactivity in the near periphery of fibrillary (ThS-positive) and Abeta-immunoreactive extracellular plaques. This labelling pattern was consistently detected irrespective of the brain region (hippocampus and neocortex) and of plaque size. In addition, marked HTT immunoreactivity was detected in amyloid plaque-associated reactive astrocytes (Fig. 4a) but not in microglial cells (not shown). In brains of wild type mice neither punctate nor astrocytic HTT immunoreactivity was observed (Fig. 4a).

In order to reveal whether HTT association with amyloid plaques directly reflects its stimulated aggregation by Abeta peptides, HTT aggregation assays in the absence or presence of 1 μM Abeta (1–42) were performed. These assays revealed stimulated HTT aggregation kinetics in the presence of Abeta (Fig. 4b). While the HTT aggregation rate increased significantly in the presence of Abeta peptides, no shortening of the lag phase was observed (Fig. 4b). This indicates that Abeta peptides increase the efficiency of the polymerization event while they do not accelerate the nucleation process. The co-aggregation of HTT with Abeta in fibrils was also demonstrated by transmission electron microscopy (Fig. 4c).

**Fig. 5:**
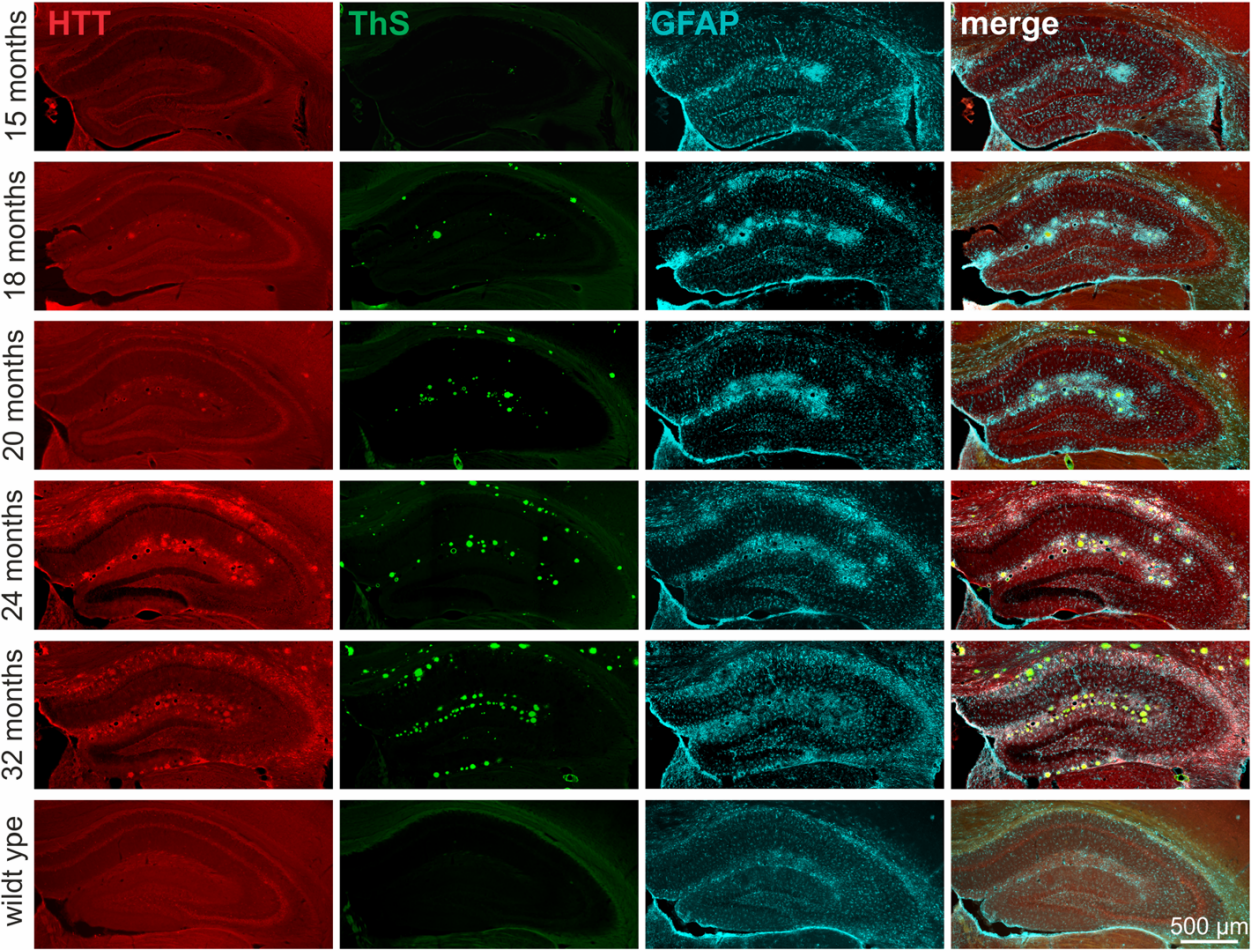
Triple fluorescent labelling of HTT (red), fibrillary Abeta aggregates (ThS, green) and reactive astrocytes (GFAP, blue) in hippocampus of Tg2576 mice and wild type control. ThS plaque labelling as well as HTT and GFAP immunoreactivity in Tg2576 mice increased in an age-dependent manner

Next, we sought to investigate whether the accumulation of endogenous HTT near Abeta plaques and its induction in reactive astrocytes are age-dependent and increase along with plaque load. Therefore, coronal brain sections of APP-transgenic mice at the postnatal age of 15, 18, 20, 24 and 32 months, respectively, were used for immunocytochemical triple labelling of HTT, fibrillary Abeta plaques (ThS) and reactive astrocytes (GFAP). We observed an age-dependent increase in hippocampal HTT immunoreactivity, which was paralleled by increases in plaque load and GFAP immunoreactivity (Fig. 5). In negative control experiments without primary antibodies, no labelling for HTT and GFAP was generated (not shown). Additionally, in wild type mice no ThS-positive deposits were detected and HTT and GFAP labellings were comparable to young Tg2576 mice (Fig. 5).

The presence of astrocytic HTT immunoreactivity in hippocampus of Tg2576 mice is particularly interesting and may result from astrocytic HTT expression and/or uptake of HTT released from neurons. In order to investigate the capability of astrocytes to express HTT, Htt mRNA and HTT protein expression were analyzed by RT-qPCR and by immunocytochemistry in primary mouse neuronal and astrocytic cultures. The purity of neuronal and astrocytic cultures was demonstrated by the detection of the neuronal marker NeuN and the astrocytic marker GFAP by RT-PCR and by immunocytochemistry (Additional file 1: Figure S2). These data clearly show the absence of neurons in astrocytic cultures and vice versa.

**Fig. 6:**
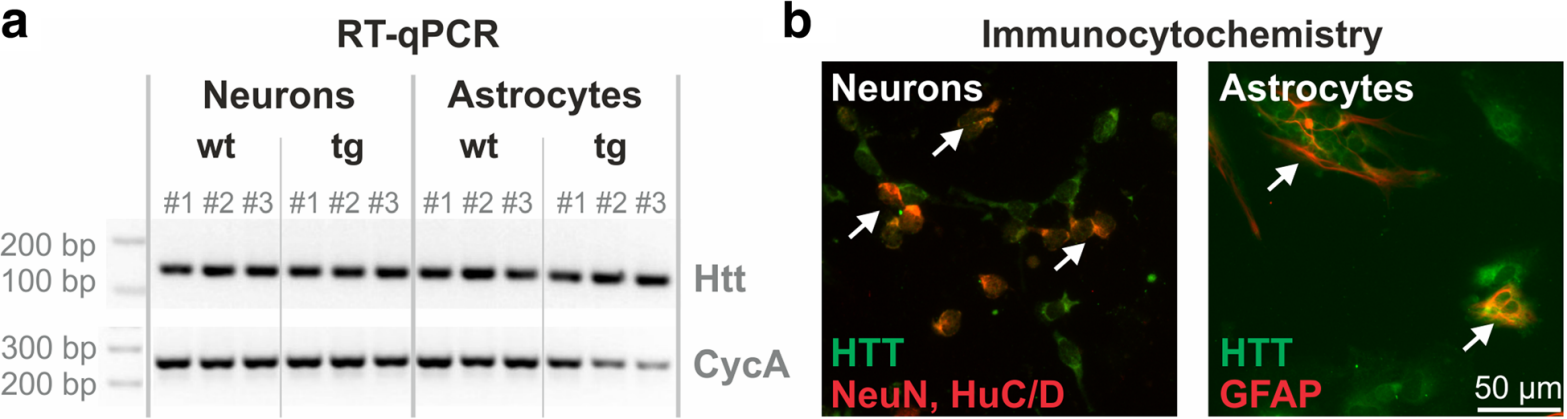
RT-qPCR and immunocytochemistry for HTT expression in mouse primary neuronal and astrocytic cultures. **a** In the upper lane the RT-qPCR products obtained with Htt primer pair #1 at the calculated product size of 152 bp are shown for primary neurons and astrocytes derived from three wild type (wt) and three Tg2576 (tg) mice. Note the similar expression levels for Htt mRNA in wt and tg neuronal and astrocytic cultures. The amount of Htt-specific PCR product was normalized to the expression of the housekeeping gene Cyclophilin A (CycA) (lower lane). **b** Immunocytochemistry reveals the presence of HTT protein (green fluorescence) in primary neurons (left) and primary astrocytes (right) as indicated by arrows. Neurons were identified by NeuN, HuC/D labelling (red fluorescence) and astrocytes were marked by GFAP immunocytochemistry (red fluorescence)

The analysis of RT-qPCR products by gel electrophoresis demonstrated the expected product size and similar HTT expression levels in primary neurons and astrocytes of both, wild type and Tg2576 origin (Fig. 6a). C_T_ mean values for each cell type, genotype and applied primer pair were calculated and are shown in Table 3 with the corresponding ΔC_T_ value. The calculated ΔC_T_ values were further taken to perform relative quantification by comparing the values among each other, obtaining ΔΔC_T_. Subsequently, based on ΔΔC_T_ values, the factor of difference between the two investigated samples was calculated (Table 4). The relative quantification revealed that both cell types investigated express Htt mRNA at comparable levels with small fluctuations. In conclusion, by means of Htt RT-qPCR we were able to validate that primary astrocytes of wild type and Tg2576 background do express Htt mRNA in addition to primary neurons.

**Table 3:** Calculated C_T_ means for each sample and primer pair with corresponding ΔC_T_ value

Sample	C_T_ mean (cyclophilin)	C_T_ mean (Htt #1)	ΔC_T_ (Htt #1)	C_T_ mean (Htt #4)	ΔC_T_ (Htt #4)
Neurons wt	13.53	16.30	2.7	16.75	3.22
Neurons tg	13.82	16.22	2.4	17.41	3.59
Astrocytes wt	11.98	14.88	2.90	15.80	3.82
Astrocytes tg	12.06	15.26	3.21	16.1	4.05

*wt* wild type, *tg* transgenic Tg2576

**Table 4:** Comparison of C_T_ means with ΔΔC_T_ method

Compared samples	ΔΔC_T_ (Htt #1)	Factor	ΔΔC_T_ (Htt #4)	Factor
Neurons wt – tg	−0.387	1.30	0.37	0.78
Astrocytes wt – tg	0.30	0.81	0.23	0.85
Neurons wt – astrocytes wt	0.13	0.92	0.6	0.66
Neurons tg – astrocytes tg	0.81	0.57	0.46	0.73

*wt* wild type, *tg* transgenic Tg2576

The data on Htt mRNA expression are supported by immunocytochemical labelling of HTT in mouse primary neurons and astrocytes demonstrating neuronal and astrocytic HTT protein expression (Fig. 6b). Interestingly, primary astrocytes from wild type mice expressed Htt mRNA and HTT protein whereas in brain of adult mice astrocytic HTT immunoreactivity was only observed in transgenic mice and associated with amyloid pathology (Fig. 4).

## Discussion

### Expression of endogenous HTT in mouse brain

In HD, the number of CAG repeats of the HTT gene determines the age of disease onset in a fully dominant fashion [47]. When assessing the neuronal populations affected by pathogenic HTT protein aggregation, it is very likely that neurons with abundant HTT expression are more affected than neurons expressing low levels of HTT or no HTT at all. Thus, the defined cellular HTT expression levels might be decisive for the neuron type-specific pathology. From that point of view it is surprising that no detailed analyses on the neuron type-specific endogenous HTT expression patterns in brains of frequently used experimental animal species exist. There is only an early report showing HTT immunoreactivity in the cytoplasm of neurons in rat neocortex and in human cerebellar Purkinje and neocortical neurons as well as neurons of caudate nucleus [17].

Therefore, we here comprehensively analyzed the brain region- and neuron type-specific expression of endogenous HTT in brains of three mouse lines, Wistar rat and hamster using a monoclonal rabbit antibody (EP867Y) that binds to the HTT mid-region. The specificity of this antibody is validated by Western blot analysis of HTT knock-out cells analyzed along with wild type cells (Product information sheet: https://www.abcam.com/huntingtin-antibody-ep867y-ab45169.html). Furthermore, in the transgenic BACHD HTT mouse model we demonstrate much stronger EP867Y labelling than in wild type mouse brain tissue (Additional file 1: Figure S1b). In addition, the specificity of the EP867Y antibody was demonstrated in a number of recent publications [32, 56, 75].

Interestingly, in the striatum of all mouse lines tested, as well as in rat and hamster, strong HTT immunoreactivity stood out in cholinergic interneurons that are spared in HD [20]. In contrast, in DARPP-32-positive striatal medium-spiny neurons, which are known to be affected in HD [29], significant HTT-labelling with the high threshold selected here was missing. However, also in human brain large striatal neurons that are spared in HD express HTT [64] and deficits in cholinergic markers such as acetylcholinesterase and vesicular acetylcholine transporter without accompanying cell loss have been reported in HD affected subjects [1, 73].

We detected ubiquitous low level HTT immunoreactivity throughout the brain but a highly selective enrichment of HTT in cholinergic neurons of the basal forebrain and amygdala, and in particular in cholinergic neurons of cranial nerve nuclei. In addition, prominent HTT expression was revealed in Ucn-1-positive EWN neurons and represented the only non-cholinergic neuronal population with abundant HTT expression identified in the present study. These observations indicate that not only neurons in striatum, but also defined neuronal populations in midbrain and brain stem may be affected by the pathogenic mechanisms resulting from polyQ extension of the HTT protein in HD. Indeed, in HD patients HTT protein inclusions were demonstrated in cranial nerve nuclei III (oculomotor nerve), IV (trochlear nerve), V (trigeminal nerve), VI (abducens nerve), VII (facial nerve), VIII (vestibulocochlear nerve), IX (glossopharyngeal nerve), X (vagal nerve) and XII (hypoglossal nerve) [63]. This is in good accordance with our observation of prominent HTT expression in mouse, rat and hamster brain and would explain some clinical symptoms, (i.e. oculomotor and vestibular deficits) that cannot solely be attributed to degeneration of striatal neurons. Interestingly, HTT-rich vestibular projection neurons predominantly terminate on HTT-negative medium spiny neurons in dorsolateral striatum [78], where significant neurodegeneration in HD occurs. Thus, in addition to local HTT expression, an indirect pathogenic HTT effect in striatum via innervating fibers should be considered based on the evidence presented here.

We would like to note that this pattern of HTT expression was not recapitulated by other HTT antibodies such as 2B7 and 2166. One possible explanation for this is HTT cleavage by different proteases to generate numerous fragments [44, 57] and the adoption of different conformational states of the HTT protein [65, 69]. The epitope of the EP867Y antibody is within amino acids 550 and 650 of HTT and corresponds to residues specific to the apopain (caspase-3) cleavage site [27, 87]. Thus, this antibody appears to be unique and will allow monitoring distinct HTT expression related to cholinergic nuclei.

From the pattern of endogenous HTT expression in mouse, rat and hamster brain, one would predict that under conditions of pathogenic HTT expression the corresponding neuronal functions such as the reward system (VP, CPu, Tu, PPT, Amy), memory processing and decision making (Amy), arousal and attention (PPT), generation of REM sleep (LDT), pupil constriction and eye movement (EWN, oculomotor nerve nucleus, nucleus abducens), parasympathetic vagal functions (dorsal motor nucleus vagus) and general sensory and motor functions (facial nucleus, Pr5, Mo5, PPT, MVePC, ambiguus nucleus, hypoglossal nucleus) are affected. Indeed, the clinical features of HD such as progressive motor dysfunction, cognitive decline and psychiatric disturbance [86] could be – at least partially – related to dysfunction or death of these neuronal populations. Typical motor dysfunctions in HD include chorea, dystonia, progressive bradykinesia, rigidity and incoordination. In addition, many patients have substantial cognitive or behavioral disturbances before onset of diagnostic motor signs [54]. Interestingly, deep brain stimulation of the PPT might ameliorate gait and postural difficulties in PD subjects [3, 79]. In addition, a significant cholinergic innervation of the striatum and nucleus accumbens arises from brainstem LDT and PPT nuclei [14]. Thus, when affected during HD pathogenesis, dysfunction and/or degeneration of these neurons may contribute to striatal cholinergic deficits. Indeed, targeting the cholinergic system has been proposed as novel HD therapy [18] and may compensate deficits in numerous cholinergic nerve nuclei with high HTT expression levels. This may also apply to the hypoglossal nerve nucleus which provides innervation of extrinsic and intrinsic muscles of the tongue and, therefore, accounts for the prominent dysphagia and disturbance of speech in HD patients [48, 53, 62].

### Subcellular localization

Neurons with highly abundant HTT immunoreactivity displayed cytoplasmic localization of HTT. This is consistent with biochemical and electron microscopic analyses which demonstrated the presence of HTT in neuronal cytoplasm and an association with vesicle membrane proteins [17]. In the cytoplasm, full length HTT seems to be particularly involved in intracellular transport processes and vesicle trafficking, as it interacts with microtubules and clathrin-coated vesicles [24, 36, 82]. In neurons with lower endogenous HTT expression levels, we also observed faint immunoreactivity in the nucleus. This is in line with reported co-immunoprecipitates with the carboxy-terminal binding protein, known to be a transcriptional co-repressor in the nucleus [43]. An amino-terminal HTT fragment, however, has also been shown to interact with a variety of proteins important for nuclear function, like p53 [77] and nuclear receptor co-repressor protein [5].

### Transgenic HD models

In several transgenic mouse models of HD, mutant HTT is randomly inserted within the genome and its expression is typically driven by neuron-specific promoters other than the endogenous HTT promoter. Most of these mice develop ubiquitinated nuclear HTT inclusions but do not exhibit the full neuropathological phenotype of human HD brains [83]. In particular, brain regions affected by neuronal loss differ from that in HD and the effects on oligodendrocytes and astrocytes in the human brain are not recapitulated in transgenic mice [83]. On the other hand, in *knock in* mouse models of HD, transgene expression is driven by the endogenous mouse HTT promoter and should lead to pathology in brain regions identified by us as highly HTT expressing. Interestingly, the manifestation of HD-typical pathology including brain atrophy, striatal neuronal loss, severe motor phenotype, weight loss and premature death depends on the expression of N-terminal HTT fragments as present in the R6/2 model expressing exon 1 of the HTT protein under the control of human HTT promoter [8, 49]. Unfortunately, the more caudal brain regions containing cholinergic cranial nerve nuclei identified by us as strongly immunoreactive for endogenous HTT were not included in the analyses. Thus, we strongly recommend the analyses of these structures and would predict the detection of pathological alterations like HTT protein aggregates and astrogliosis in these clearly defined nuclei.

### Protein cross seeding

An important aspect of the present work was the analyses of a potential HTT protein cross seeding by Abeta aggregates in brains of transgenic mice with amyloid pathology. As the amyloid pathology in the Tg2576 mouse model used is restricted to neocortex and hippocampus, we focused on HTT aggregation in proximity to amyloid plaques in these brain regions. We observed an extracellular halo of HTT immunoreactivity around amyloid plaques labelled with the Abeta-specific antibody 4G8 or by the fibril intercalating dye ThS, respectively. Such protein cross seeding events are known to occur for proteins affected by a particular disease, such as Abeta and tau proteins in AD [28], but also for proteins from different clinical entities such as Abeta and α-synuclein proteins affected in AD and PD [23, 46, 52, 55, 80], respectively. We here demonstrate for the first time that also the endogenous HTT proteins can cross-seed with Abeta aggregates. This process has a clear age-related component and Abeta plaque-associated HTT immunoreactivity in hippocampus increases with plaque load. Since Abeta plaques also contain non-Abeta peptides that could be responsible for HTT recruitment, we directly tested the aggregation of HTT by Abeta (1-42) in ThT aggregation assays. We demonstrate that Abeta peptides increase the efficiency of the polymerization event but have no effect on the nucleation process. The nucleation of polyQ HTT was reported to underlie a different mechanism than nucleation of other amyloid peptides [38]. PolyQ fibrils of HTT do not reveal a typical β-sheet structure but rather assemble into α-helical structures and oligomers which interfere with polyQ sequences and facilitate the formation of amyloid nuclei [58]. A similar effect could occur in the presence of Abeta peptides generating conditions that favor β-sheet structure and thus result in higher aggregation rates [74]. The biological significance and pathogenic potential of these co-aggregates is not yet clear but they might represent novel therapeutic targets. It has been recently shown in an AD mouse model that targeting one pathogenic protein by passive immunization also attenuates the deposition of other pathogenic protein aggregates, most likely by a mechanism involving activation of the complement system and microglia [13].

Currently, no data are available on cross seeding of HTT to Abeta plaques in AD brains. However, there are several reports analyzing Abeta pathology in HD brains [15, 40, 76]. Based on morphological amyloid and tau staging in autopsy cases of HD, a rare co-existence of HD and AD was reported, although initial neuropathological stages of AD were found to be present early in HD patients [40]. On the other hand, in elderly HD subjects with dementia, a high proportion of subjects displayed tau and amyloid pathology, suggesting that co-occurrence of AD may contribute to cognitive decline in elderly HD patients [15]. Moreover, a co-occurrence of mixed proteinopathies involving oligomerization and aggregation of Tau, α-synuclein and TDP-43 in late stage HD was demonstrated [76], indicating common mechanisms of pathological protein aggregation in different neurodegenerative diseases.

### HTT expression by astrocytes

In brain sections of Tg2576 mice used for the analyses of HTT protein cross seeding to amyloid plaques we surprisingly observed HTT immunoreactivity in reactive astrocytes in proximity to Abeta plaques. This robust astrocytic HTT immunoreactivity raises the question whether HTT produced by and released from neurons is taken up by astrocytes or whether it is expressed by these glial cells. In order to address this issue, primary neuronal and glial cell cultures from APP-transgenic Tg2576 mice were established and analyzed for Htt mRNA and protein expression. Both, primary neurons and astrocytes were shown to express Htt mRNA and protein. This is in agreement with immunohistochemical labellings of brain slices and supportive of de novo synthesis of HTT by astrocytes, rather than uptake of neuron-derived HTT. However, in contrast to the labelling of mouse brain sections, also astrocytes derived from primary cultures of wild type mice expressed HTT indicating that astrocytic HTT expression can generally be induced under conditions of astrocytic activation and independent of Abeta pathology. HTT immunoreactive astrocytes were also detected in the caudate and putamen of HD subjects [72] and in a transgenic HTT mouse model [71]. The intentional and specific mutant HTT expression by astrocytes was demonstrated to cause age-dependent neurological symptoms including body weight loss, motor function deficits, increased susceptibility to glutamate-induced seizures and early death [6, 7]. Moreover, the mutant HTT expression by cultured primary astrocytes was shown to induce cell death of co-cultured wild type neurons [71]. On the other hand, reduction of astrocytic mutant HTT expression slows disease progression in the BACHD conditional HD mouse model and improves motor and psychiatric-like phenotypes [89]. Thus, there is solid evidence to conclude that astrocytic HTT expression contributes to neurodegeneration in HD.

## Conclusions

We demonstrate a selective enrichment of physiological, endogenous HTT in cranial neve nuclei in mouse, rat and hamster brain. Under clinical conditions of pathogenic HTT variant expression, the functions of these neuronal populations might be compromised. Indeed, clinical symptoms of HD patients not related to dysfunction of striatal medium spiny neurons can be partly attributed to pathogenesis in cranial nerve nuclei. We also demonstrate for the first time an association of endogenous HTT to amyloid plaques and the expression of HTT by reactive astrocytes in a transgenic animal model of AD. These data are in line with cross-disease mechanisms of pathogenic protein co-aggregation present under conditions of HD and AD.

## Additional file

Additional file 1: Supplementary Information. (PDF 385 kb)

## Abbreviations

AD: Alzheimer’s disease
Amb: Ambiguus nucleus
Amy: Amygdala
APP: Amyloid precursor protein
BSA: Bovine serum albumin
ChAT: Choline acetyltransferase
CPu: Caudate putamen
DAB: 3,3′-diaminobenzidine
DARPP-32: Dopamine and cAMP regulated phosphoprotein 32 kDa
EWN: Edinger-Westphal nucleus
GFAP: Glial fibrillary acidic protein
HD: Huntington’s disease
HDB: Horizontal diagonal band of Broca
HTT: Huntingtin
LC: Locus coeruleus
LDT: Laterodorsal tegmental nucleus
Mo5: Motor trigeminal nucleus
MS: Medial septum
MVePC: Parvicellular medial vestibular nucleus
Nbm: Nucleus basalis magnocellularis
PBS: Phosphate-buffered saline
PCRtA: Parvicellular reticular nucleus
PD: Parkinson’s disease
PPT: Pedunculopontine tegmental nucleus
TBS: Tris-buffered saline
TH: Tyrosine hydroxylase
ThS: Thioflavin S
Tu: Olfactory tubercle
Unc-1: Urocortin-1
VDB: Vertical diagonal band of Broca
VP: Ventral pallidum
