# Supplementary material for: Endogenous mouse huntingtin is highly abundant in cranial nerve nuclei, co-aggregates to Abeta plaques and is induced in reactive astrocytes in a transgenic mouse model of Alzheimer’s disease
Source: Acta Neuropathol Commun. 2019 May 20;7:79. doi: 10.1186/s40478-019-0726-2 (PMC6526682; doi:10.1186/s40478-019-0726-2)
Supplement: Supplementary file 1 — Supplementary Information. (PDF 385 kb) [file 40478_2019_726_MOESM1_ESM.pdf]

Supplementary Information to

Endogenous mouse Huntingtin is highly abundant in cranial nerve nuclei, co-aggregates to Abeta plaques and is induced in reactive astrocytes in a transgenic mouse model of Alzheimer's disease

by: Maike Hartlage-Rübsamen et al.

HTT antibody dilution testing and specificity

In initial experiments, a dilution series of the primary EP867Y HTT antibody ranging from 1:500 to 1:4,000 was tested for immunohistochemical labellings on wild type mouse brain sections. Based on the signal-to noise ratio and the marked appearance of neurons with highly abundant HTT expression a 1:4,000 dilution of the primary antibody was used for the subsequent serial HTT stainings. However, it should be noted that a ubiquitous HTT immunoreactivity was observed at higher antibody concentrations (**Suppl. Fig. 1a**). In order to validate the specificity of the EP867Y HTT antibody, a side-by-side comparison of the immunohistochemical labelling generated in wild type and in HTT overexpressing BACHD mice (Gray et al., 2013) was performed (**Suppl. Fig. 1b**). In wild type mouse brain sections of the hippocampal coronal level only faint labelling was present, whereas in BACHD mouse brain with robust HTT overexpression strong HTT immunoreactivity in neocortex and hippocampus was observed (**Suppl. Fig. 1b**).

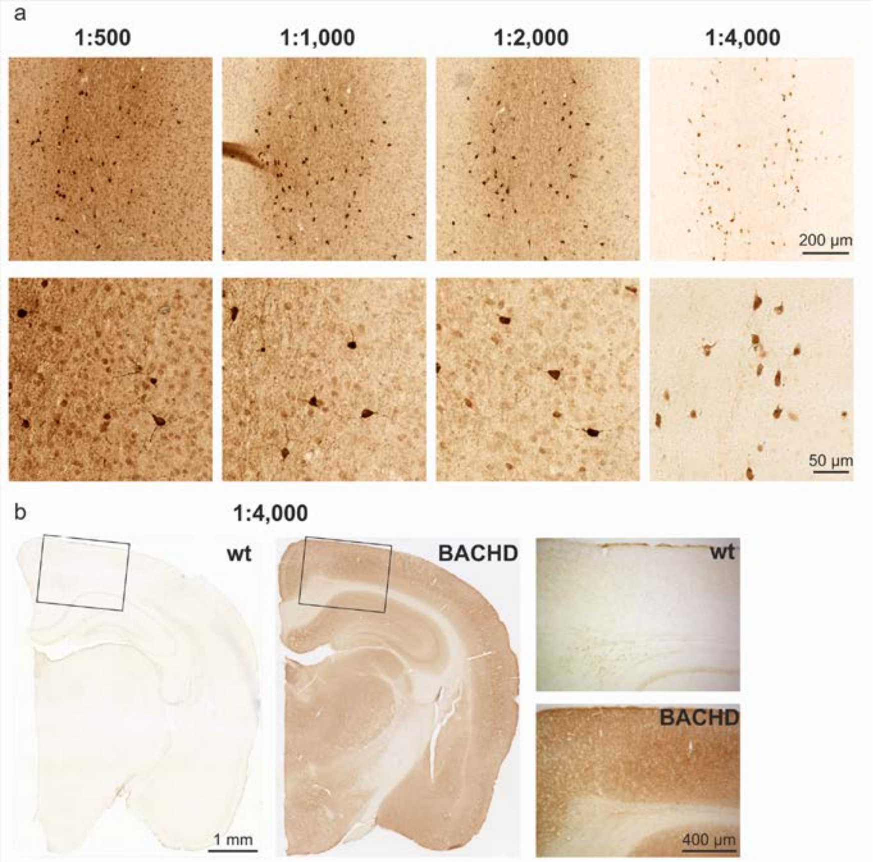

Suppl. Fig. 1:
(a) Immunohistochemical labelling of HTT using the EP867Y antibody at different dilutions as indicated ranging from 1:500 to 1:4,000 on mouse brain sections on the basal forebrain coronal cutting level. Note the robust HTT expression by neurons of the medial septum and vertical diagonal band and the additional ubiquitous low levels of HTT immunoreactivity detected when using higher antibody concentrations.
(b) Comparison of the immunohistochemical signal generated by the EP867Y antibody (1:4,000) in wild type (wt) and HTT-transgenic BACHD mouse brain sections with robust HTT overexpression. Note the much stronger overall HTT immunoreactivity in BACHD mouse brain.

Purity of primary neuronal and astrocytic cultures

In order to demonstrate the purity of primary neuronal and astrocytic cultures, RT-PCR to amplify NeuN mRNA and GFAP mRNA and immunocytochemistry to detect the NeuN, HuC/D and GFAP were performed. While GFAP mRNA and protein were virtually absent form neuronal cultures, the neuron-specific markers NeuN and HuC/D were not detected in astrocytic cultures (**Suppl. Fig. 2**). Collectively, these data demonstrate the high purity of both primary cell cultures.

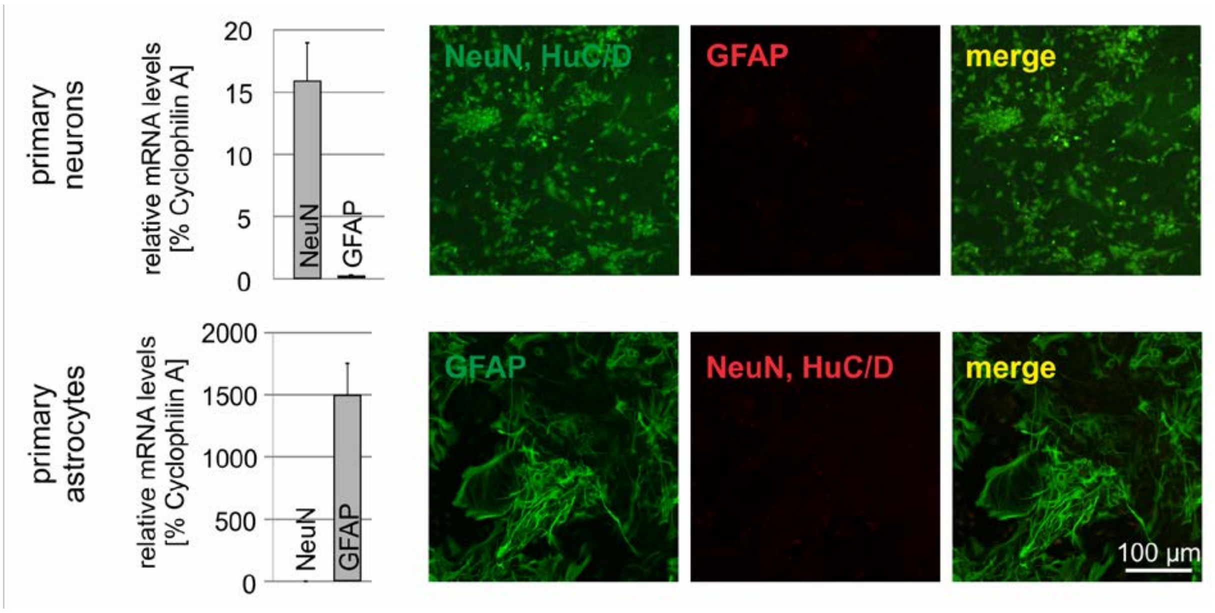

Suppl. Fig. 2: *Left:* Cyclophilin A-normalized qRT-PCR for the neuronal marker NeuN and the astrocytic marker GFAP in primary neurons and primary astrocytes as indicated. *Right:* Immunocytochemistry for neuronal markers NeuN and HuC/D and for the astrocyte marker GFAP in primary neurons and primary astrocytes as indicated. Note the absence of neuronal markers in astrocytes and *vice versa*.

Htt primer selection and characterization

Primers specific for Htt mRNA were designed with NCBI Primer Blast software under the following premises: GC-content between 50% and 60%, self-complementarity under 5 to avoid formation of internal secondary structures, similar melting temperature (T_m) of forward (fw) and reverse (re) primer, product length under 300 bp and the target region should span two exons to exclude amplification of genomic DNA. Four designed primer pairs and a primer pair published by Szlachcic et al. (2015) (#3) were tested (**Suppl. Table 1**).

Suppl. Table 1: List of primers tested for RT-qPCR. The primers are listed with their respective sequence, melting temperature (Tm), GC content, 3' and 5' self-complementarity and product size.

**Primer**	**Sequence (5' -> 3')**	**T_m [°C]**	**GC content**	**Self-complementarity 3'**	**5'**	**Product size**
**fw #1**	AGCCACCAAGAAAGACCGTG	60.54	55.00	3.00	1.00	152 bp
**re #1**	TGACATCTGACTCCGCATCG	59.90	55.00	3.00	2.00	
**fw #2**	CAGTGACGATGCGGAGTCAG	60.80	60.00	4.00	3.00	148 bp
**re #2**	ACGCAAACTTCGAGGAGCAC	61.22	55.00	4.00	2.00	
**fw #3**	CCCTGGAAAAGCTGATGAAG	56.39	50.00	4.00	2.00	231 bp
**re #3**	CACGGTCTTTCTTGGTGGCTG	61.74	57.14	3.00	1.00	
**fw #4**	CTGCACGGCATCCTCTATGT	59.89	55.00	4.00	0.00	123 bp
**re #4**	AATGTTCACGCAGTGGGCTA	59.96	50.00	4.00	2.00	
**fw #5**	TCCAATCAGCAGCCATACCC	59.82	55.00	3.00	0.00	123 bp
**re #5**	GAAGTTGGACAGGGACAGCA	59.89	55.00	3.00	0.00	

A one-step RT-qPCR with whole brain wild type RNA as template was performed to control the specificity of primer pairs. All working steps were performed on ice using the Qiagen RT-PCR Kit. RNA of a wild type brain (2273.3 ng/µl) was diluted 1:22.73 in RNase-free water to gain a 100 ng/µl solution. This dilution was denatured at 70°C for 5 minutes in a Flex Cycler to dissolve possible secondary structures and was stored on ice until further usage. Primers (50 µM) were diluted 1:5 in *aqua dest*. to obtain a 10 µM concentration. A master mix was prepared and 18 µl of master mix were mixed with 1 µl fw primer and 1 µl re primer. The RT-qPCR was performed with a Flex Cycler according to **Suppl. Table 2**.

Samples were analyzed with a 2% agarose gel using the GeneRuler 100 bp DNA ladder as standard. The electrophoresis was run for 50 minutes at 120 V. Pictures were taken with Biostep ArgusX1 Software and colors were inverted with Adobe Photoshop CS2 Software.

Suppl. Table 2: Program for RT-qPCR with Flex Cycler.

stage		temperature [°C]	time [s]
hold stage 1		50	1800
		95	900
cycling stage:	denaturation	95	10
	primer annealing	58	45
	extension	72	45
hold stage 2		72	10
		4	∞

A one-step RT-qPCR with whole wild type brain RNA as template was performed to control for the specificity of these primers followed by agarose gel electrophoresis. Primer pairs #1 to #5 all showed one single band of the predicted size when compared to the DNA base pair ladder (**Suppl. Fig. 3a**). Based on these results and taking into account the general characteristics of each primer (**Suppl. Table 1**), primer pairs #1 and #4 were chosen for further investigation of Htt mRNA expression. The linearity of the RT-PCR under the experimental conditions was demonstrated by running a standard curve for Cyclophylin A amplification with different concentrations of total RNA (**Suppl. Fig. 3b**). The individual values calculated for wild type and for Tg2576 primary neurons and astrocytes as indicated fit well to the middle part of the standard curve (**Suppl. Fig. 3b**). In control experiments without RT, no PCR products were generated from the RNA preparations of wild type and Tg2576 mouse brains (**Suppl. Fig. 3c**). Moreover, DNAse treatment of isolated RNA did not affect RT-PCR product band intensity demonstrating the formation of PCR products from isolated RNA and not from DNA contamination (**Suppl. Fig. 3c**). The negative control (PCR without cDNA template) did not generate PCR products (**Suppl. Fig. 3c**).

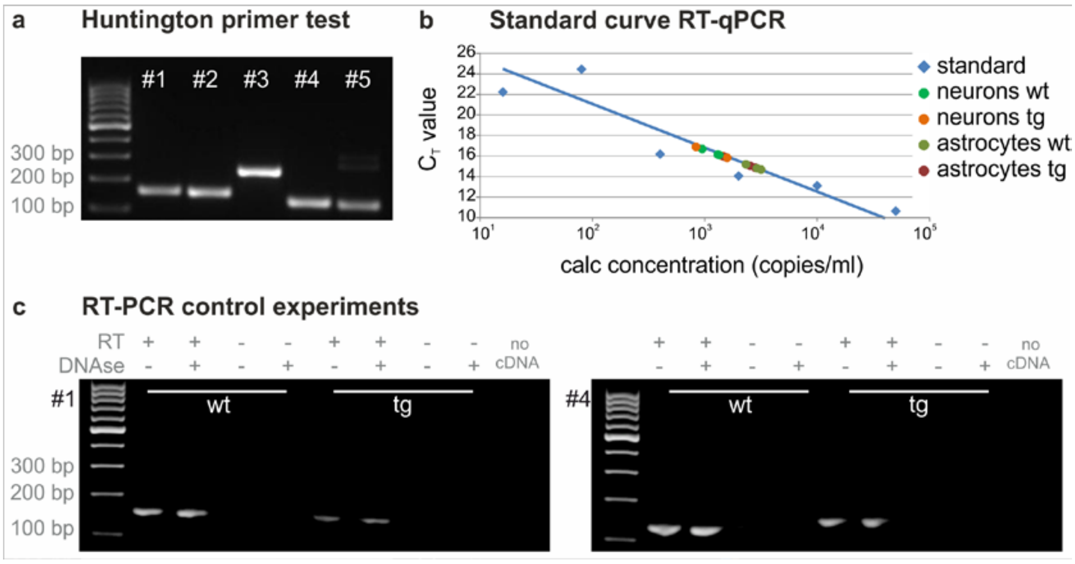

Suppl. Fig. 3: (a) Electrophoresis of PCR products for the detection of Htt mRNA with five different primer pairs (#1 to #5). S indicates the 100 bp Gene Ruler. All primer pairs show one single band with the calculated product size (see Suppl. Table 1). **(b)** Standard curve for Cyclophylin A (blue) run with different concentrations of total RNA. The individual values calculated for wild type and Tg2576 primary neurons and astrocytes as indicated fit well to the middle part of the standard curve. **(c)** Negative controls for PCR without RT and effect of DNAse treatment. Without RT, no PCR products were amplified. After DNAse digestion of isolated RNA, similar PCR products were detected, indicating their origin from mRNA, not from genomic DNA.

HTT expression in hamster brain

In order to validate our observations on distinct HTT expression made in mouse and rat brain, we also analyzed HTT expression patterns in hamster brain. In similarity to mouse brain (Fig. 2) and rat brain (Fig. 3) there was an enrichment of HTT immunoreactivity in cranial nerve nuclei and in the cholinergic basal forebrain and caudate putamen structures (**Suppl. Fig. 4**). The labelling of neocortical neurons, CA1 pyramidal neurons and dentate gyrus granule neurons in hamster recapitulated the staining pattern detected in rat brain.

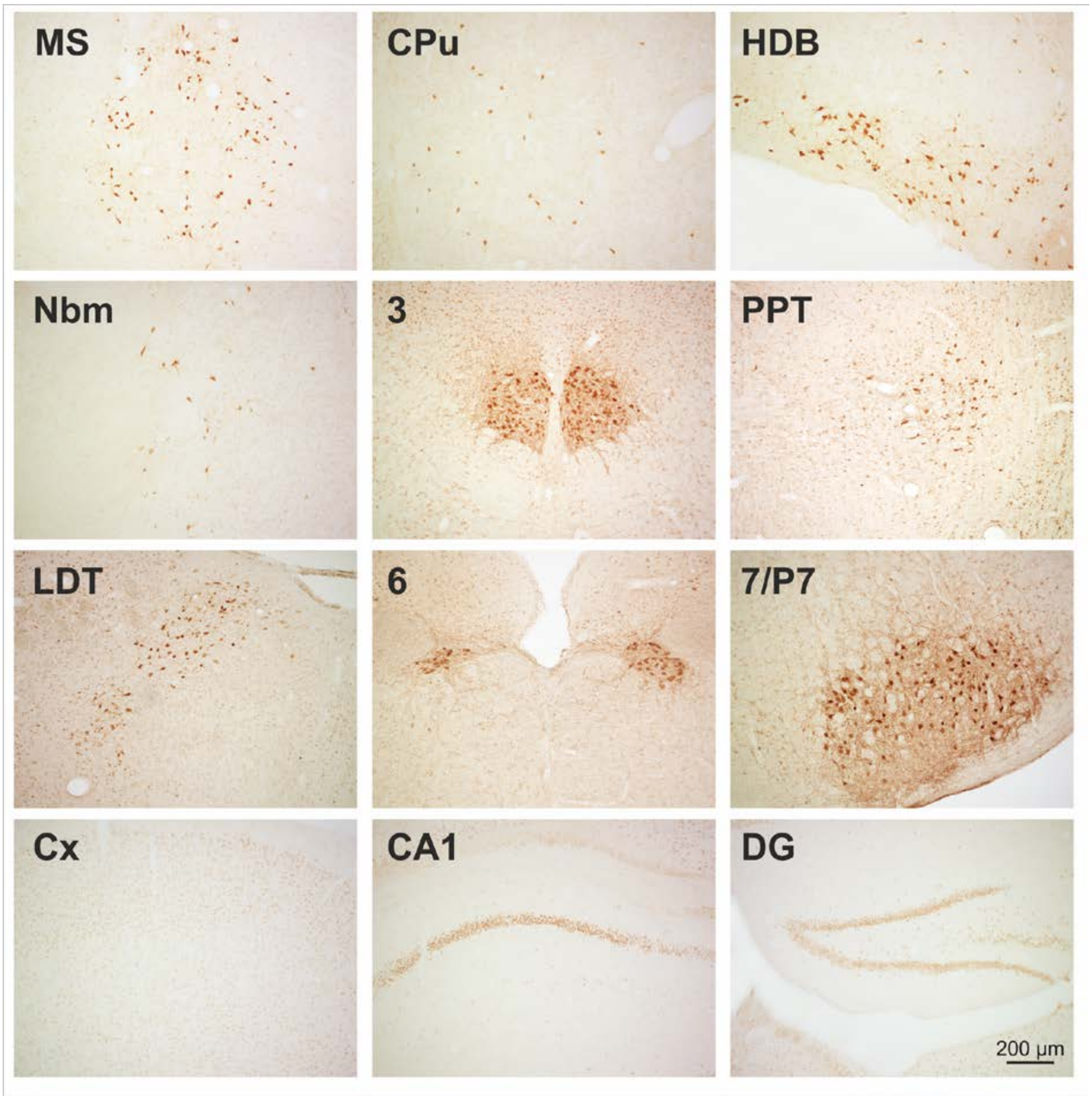

Suppl. Fig. 4: Immunohistochemical HTT labelling in coronal brain sections of hamster. Note the high abundance of HTT immunoreactivity in medial septum (MS), caudate putamen (CPu), horizontal diagonal band (HDB), nucleus basalis magnocellularis (Nbm), oculomotor nerve nucleus (3), pedunculopontine tegmental nucleus (PPT), laterodorsal tegmental nucleus (LDT), nucleus abducens (6), nucleus facialis (7) and perifacial zone (P7). In addition and in contrast to mouse brain, HTT immunoreactivity was detected in cortical neurons (Cx), and in cornu ammonis 1 (CA1) pyramidal and dentate gyrus (DG) granule neurons of the hippocampus.

References

Gray M, Shirasaki DI, Cepeda C, André VM, Wilburn B, Lu XH, Tao J, Yamazaki I, Li SH, Sun YE, Li XJ, Levine MS, Yang XW (2008) Full-length human mutant huntingtin with a stable polyglutamine repeat can elicit progressive and selective neuropathogenesis in BACHD mice. J Neurosci 28:6182–6195.

Szlachcic WJ, Switonski PM, Krzyzosiak WJ, Figlerowicz M, Figiel M (2015) Huntington disease iPSCs show early molecular changes in intracellular signaling, the expression of oxidative stress proteins and the p53 pathway. Dis Models Mech 8:1047–1057.
